# Prospective study of epigenetic alterations responsible for isolated hemihyperplasia/hemihypoplasia and their association with leg length discrepancy

**DOI:** 10.1186/s13023-021-02042-6

**Published:** 2021-10-09

**Authors:** Chang Ho Shin, Chaemoon Lim, Hwa Young Kim, Won Joon Yoo, Tae-Joon Cho, In Ho Choi, Jung Min Ko

**Affiliations:** 1 Division of Paediatric Orthopaedics, Seoul National University Children’s Hospital, Seoul National University College of Medicine, 101 Daehak-ro Jongno-gu, Seoul, 03080 Republic of Korea; 2 Department of Orthopaedic Surgery, Jeju National University Hospital, 15 Aran 13-gil, Jeju, 63241 Republic of Korea; 3 Division of Clinical Genetics, Department of Paediatrics, Seoul National University Children’s Hospital, Seoul National University College of Medicine, 101 Daehak-ro Jongno-gu, Seoul, 03080 Republic of Korea

**Keywords:** Hemihyperplasia, Hemihypoplasia, Lateralized overgrowth, Beckwith–Wiedemann syndrome, Silver–Russell syndrome, Leg length discrepancy

## Abstract

**Background:**

Hemihyperplasia and hemihypoplasia result in leg length discrepancy (LLD) by causing skeletal asymmetry. Beckwith–Wiedemann syndrome (BWS) and Silver–Russell syndrome (SRS) are opposite growth-affecting disorders caused by opposite epigenetic alterations at the same chromosomal locus, 11p15, to induce hemihyperplasia and hemihypoplasia, respectively. Because of their somatic mosaicism, BWS and SRS show a wide spectrum of clinical phenotypes. We evaluated the underlying epigenetic alterations and potential epigenotype-phenotype correlations, focusing on LLD, in a group of individuals with isolated hemihyperplasia/hemihypoplasia.

**Results:**

We prospectively collected paired blood-tissue samples from 30 patients with isolated hemihyperplasia/hemihypoplasia who underwent surgery for LLD. Methylation-specific multiplex-ligation-dependent probe amplification assay (MS-MLPA) and bisulfite pyrosequencing for differentially methylated regions 1 and 2 (DMR1 and DMR2) on chromosome 11p15 were performed using the patient samples. Samples from patients showing no abnormalities in MS-MLPA or bisulfite pyrosequencing were analyzed by single nucleotide polymorphism (SNP) microarray and *CDKN1C* Sanger sequencing. We introduced a metric named as the methylation difference, defined as the difference in DNA methylation levels between DMR1 and DMR2. The correlation between the methylation difference and the predicted LLD at skeletal maturity, calculated using a multiplier method, was evaluated. Predicted LLD was standardized for stature. Ten patients (33%) showed epigenetic alterations in MS-MLPA and bisulfite pyrosequencing. Of these, six and four patients had epigenetic alterations related to BWS and SRS, respectively. The clinical diagnosis of hemihyperplasia/hemihypoplasia was not compatible with the epigenetic alterations in four of these ten patients. No patients showed abnormalities in SNP array or their *CDKN1C* sequences. The standardized predicted LLD was moderately correlated with the methylation difference using fat tissue (r = 0.53; *p* = 0.002) and skin tissue (r = 0.50; *p* = 0.005) in all patients.

**Conclusions:**

Isolated hemihyperplasia and hemihypoplasia can occur as a spectrum of BWS and SRS. Although the accurate differentiation between isolated hemihyperplasia and isolated hemihypoplasia is important in tumor surveillance planning, it is often difficult to clinically differentiate these two diseases without epigenetic tests. Epigenetic tests may play a role in the prediction of LLD, which would aid in treatment planning.

**Supplementary Information:**

The online version contains supplementary material available at 10.1186/s13023-021-02042-6.

## Background

Leg length discrepancy (LLD) is a common orthopedic condition that can cause various problems such as scoliosis and excessive stress on hip or knee joints [1, 2]. The etiology of LLD has long been of interest to orthopedic surgeons. Hemihyperplasia or hemihypoplasia, better known as congenital hemihypertrophy or hemihypotrophy to orthopedic surgeon, results in LLD by causing skeletal asymmetry [3–8]. Hemihyperplasia/hemihypoplasia can occur as a part of a recognized clinical syndrome or in isolation. Hemihyperplasia may be caused by neurofibromatosis type 1, Klippel–Trenaunay–Weber syndrome, Proteus syndrome, or Beckwith–Wiedemann syndrome (BWS), whereas hemihypoplasia may arise from Turner syndrome or Silver–Russell syndrome (SRS) [9, 10]. The etiologies of isolated hemihyperplasia/hemihypoplasia are not well-understood [1, 3, 9]. Although the term “hemihyperplasia/hemihypertrophy” has been refined as “lateralized overgrowth” in the genetics community [11], we used the term “hemihyperplasia” in the present study to expand its readership to orthopedic surgeons who are more familiar with the term “hemihyperplasia/hemihypertrophy” than “lateralized overgrowth”.

Epigenetics refers to modification of DNA, chromatin, and associated molecules that regulates gene expression without causing alterations to the DNA sequence itself. The major epigenetic mechanisms include DNA methylation, histone modifications, and RNA-mediated processes. Genomic imprinting is an epigenetic phenomenon resulting in monoallelic expression of a gene in a parent-of-origin-specific manner. Imprinted genes are typically arranged in clusters, where their expression is controlled by differentially methylated regions (DMR) between maternal and paternal chromosomes. BWS and SRS are opposite growth-affecting genomic imprinting disorders caused by opposite epigenetic alterations at the same chromosomal locus, to induce hemihyperplasia and hemihypoplasia, respectively [12, 13]. Epigenetic alterations in a cluster of imprinted genes on chromosome 11p15 are observed in approximately 80 % of patients with BWS and 50 % of patients with SRS [12, 13].

**Fig. 1:**
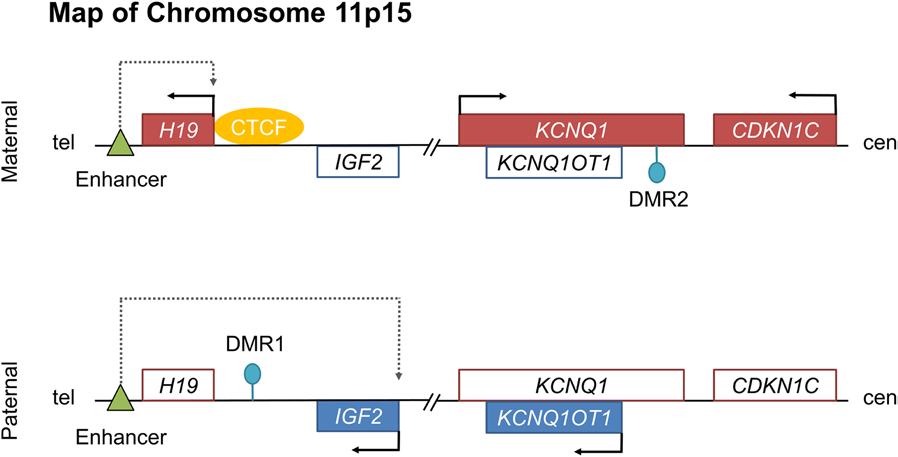
A map of chromosome 11p15. Normally, the maternal allele is methylated at differentially methylated region 2 (DMR2), and the paternal allele is methylated at differentially methylated region 1 (DMR1). Unmethylated DMR1 on the maternal chromosome permits binding of the insulator protein CTCF, which blocks access of enhancers to the *IGF2* promoter. Therefore, the maternal copy of H19 uses enhancers and is transcribed. Methylated DMR1 on the paternal chromosome prevents binding of the CTCF. Therefore, enhancers can access the *IGF2* promoter, which is transcribed. On the maternal chromosome, DMR2 is methylated, and *KCNQ1* and *CDKN1C* are transcribed. On the paternal chromosome, DMR2 is unmethylated, and *KCNQ1OT1* is transcribed. *IGF2* positively and *CDKN1C* negatively regulates cell growth and proliferation. Green triangles indicate enhancers. Lollipops indicate methylated DMR. Genes normally expressed from the maternal chromosome are depicted as red boxes, and genes normally expressed from the paternal chromosome are depicted as blue boxes. Arrows indicate the orientation of transcription. Tel = telomere. Cen = centromere

**Fig. 2:**
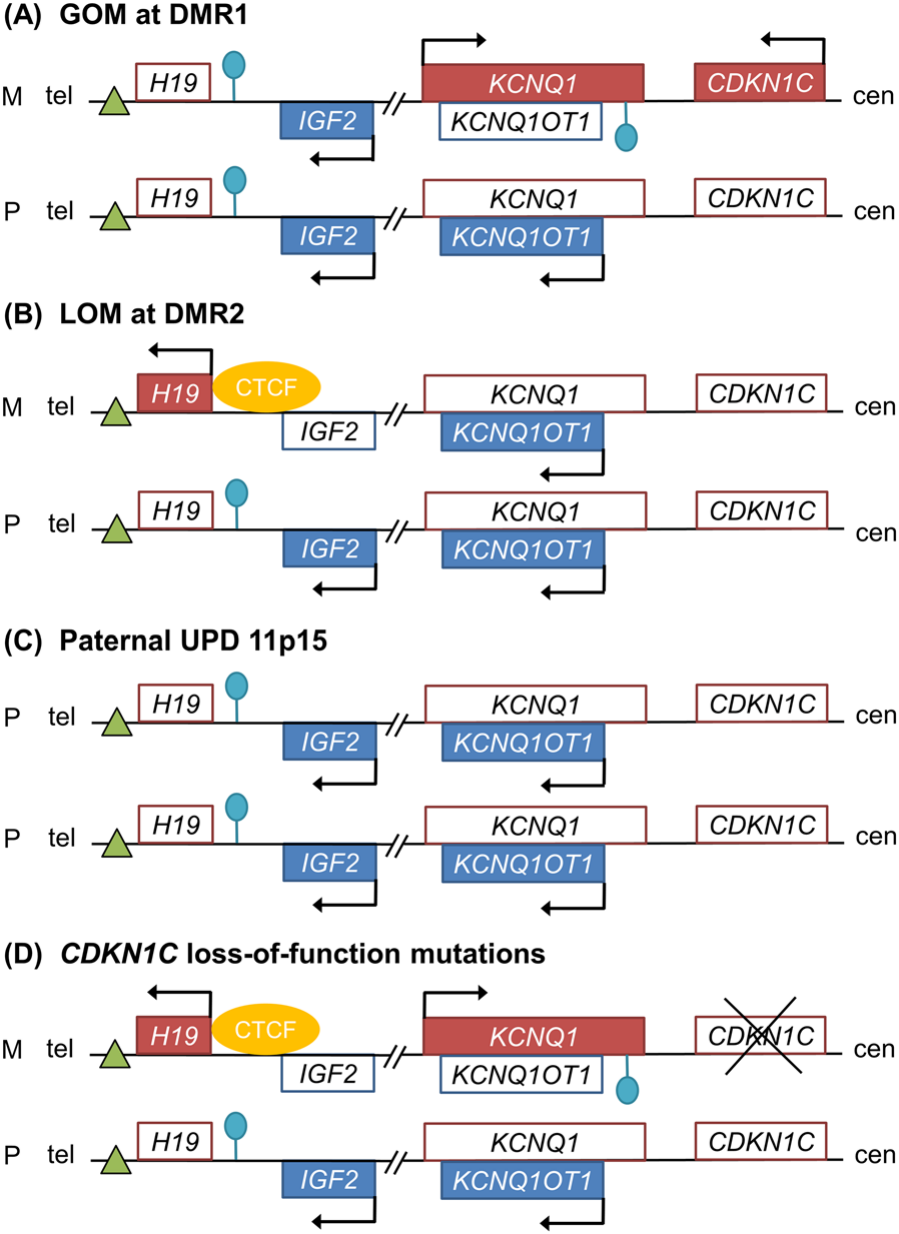
Molecular mechanism of Beckwith–Wiedemann syndrome. **a** Gain of methylation (GOM) at differentially methylated region 1 (DMR1) on the maternal chromosome results in downregulation of *H19* and expression of *IGF2*. **b** Loss of methylation (LOM) at differentially methylated region 2 (DMR2) on the maternal chromosome results in downregulation of *KCNQ1* and *CDKN1C* and expression of *KCNQ1OT1*. **c** Paternal uniparental disomy (UPD) occurs when a patient has two copies of the paternal chromosome and none of the maternal chromosome. Paternal UPD 11p15 results in overexpression of *IGF2* in addition to downregulation of *KCNQ1* and *CDKN1C.* **d** *CDKN1C* loss-of-function mutations on the maternal chromosome also result in Beckwith–Wiedemann syndrome. Green triangles indicate enhancers. Lollipops indicate methylated imprinting centers. Genes normally expressed from the maternal chromosome are depicted as red boxes, and genes normally expressed from the paternal chromosome are depicted as blue boxes. Arrows indicate the orientation of transcription. M = maternal. P = paternal. Tel = telomere. Cen = centromere

**Fig. 3:**
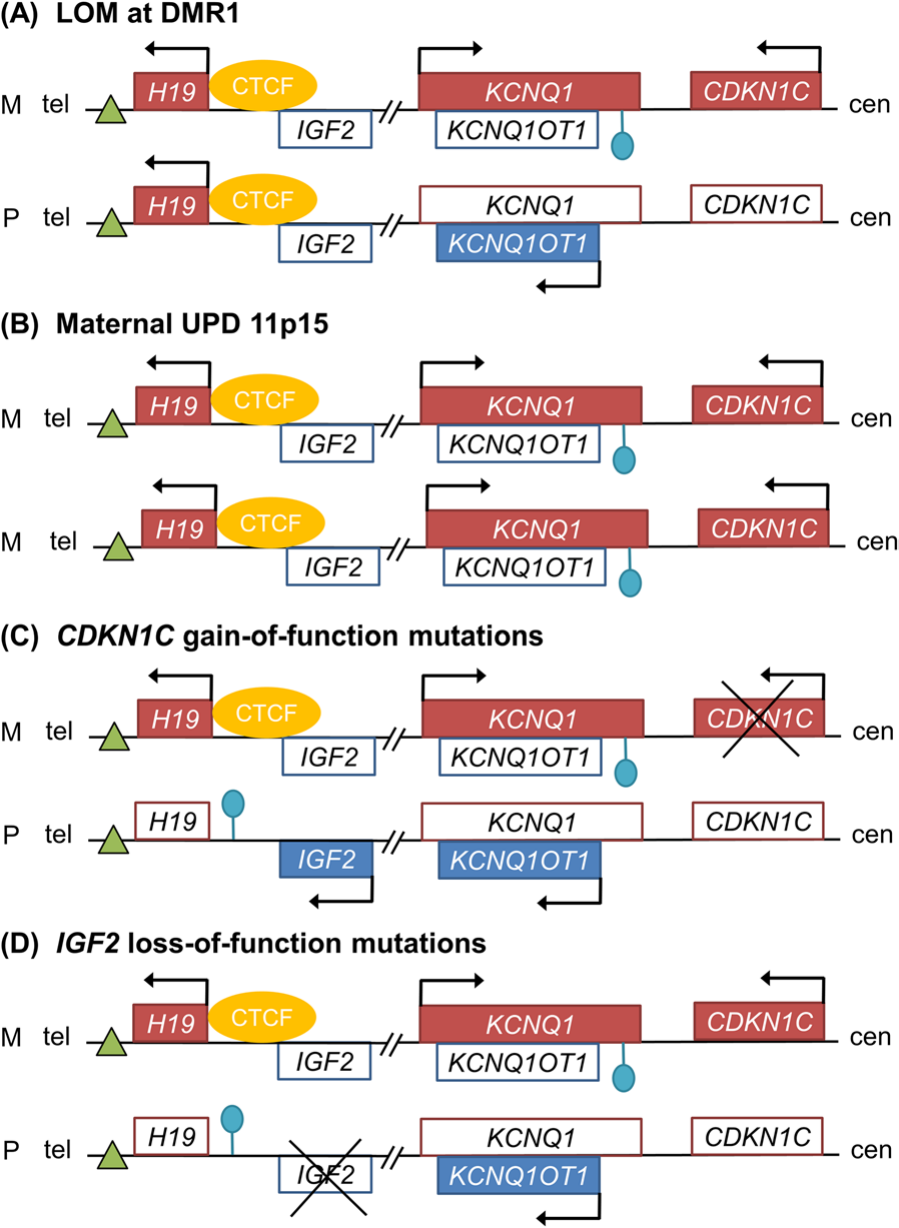
Molecular mechanism of Silver–Russell syndrome. **a** Loss of methylation (LOM) at differentially methylated region 1 (DMR1) on the paternal chromosome results in expression of *H19* and downregulation of *IGF2*. **b** Maternal uniparental disomy (UPD) occurs when a patient has two copies of the maternal chromosome and none of the paternal chromosome. Maternal UPD 11p15 results in downregulation of *IGF2* in addition to overexpression of *KCNQ1* and *CDKN1C*. **c** *CDKN1C* gain-of-function mutations on the maternal chromosome and **d** *IGF2* loss-of-function mutations on the paternal chromosome also result in Silver–Russell syndrome. Green triangles indicate enhancers. Lollipops indicate methylated imprinting centers. Genes normally expressed from the maternal chromosome are depicted as red boxes, and genes normally expressed from the paternal chromosome are depicted as blue boxes. Arrows indicate the orientation of transcription. M = maternal. P = paternal. Tel = telomere. Cen = centromere

Normally, somatic growth is balanced between two imprinted genes on 11p15, *CDKN1C* and *IGF2* that negatively and positively regulates cell proliferation, respectively [14]. The former is transcribed from the maternal chromosome, whereas the latter is transcribed from the paternal chromosome (Fig. 1) [14]. BWS/SRS can occur by (1) gain of methylation (GOM) or loss of methylation (LOM) at DMR1 or DMR2 on 11p15, (2) 11p15 uniparental disomy (UPD) which refers to the inheritance or presence of two copies of a chromosome, or part of a chromosome, from one parent and no copies from the other parent, (3) germline mutations in *CDKN1C* or *IGF2*, or (4) 11p15 copy number variation [14–19]. GOM at DMR1, LOM at DMR2, paternal UPD 11p15, and *CDKN1C* loss-of-function mutations cause overgrowth through overexpression of *IGF2* and/or downregulation of *CDKN1C*, eventually resulting in BWS (Fig. 2) [14, 16, 19]. LOM at DMR1, maternal UPD 11p15, *CDKN1C* gain-of-function mutations, and *IGF2* loss-of-function mutations cause undergrowth through downregulation of *IGF2* and/or overexpression of *CDKN1C*, resulting in SRS (Fig. 3) [15, 17, 18].

**Fig. 4:**
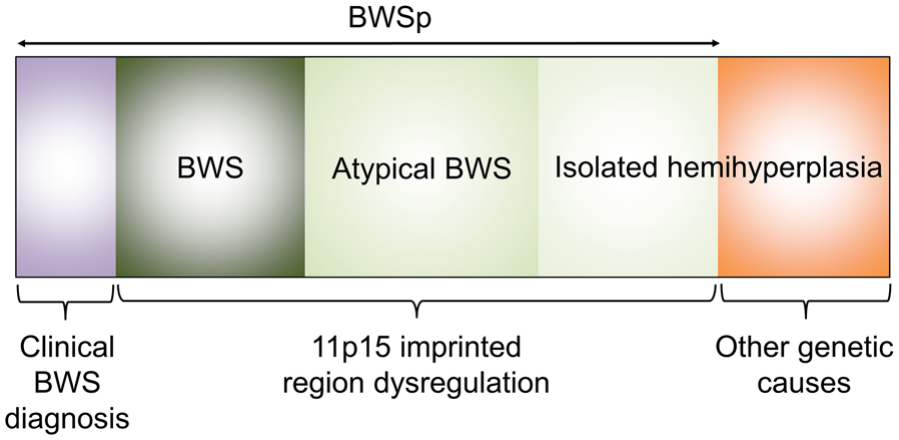
Beckwith–Wiedemann spectrum. The Beckwith–Wiedemann spectrum (BWSp) includes patients who meet the clinical diagnostic criteria of Beckwith–Wiedemann syndrome (BWS) with or without (epi)genetic alterations at the BWS locus on chromosome 11p15, patients with fewer features of BWS and (epi)genetic alterations at 11p15, and patients with isolated hemihyperplasia and (epi)genetic alterations at 11p15. (figure modified from Brioude F et al. Nat Rev Endocrinol. 2018)

Somatic mosaicism refers to the occurrence of two genetically distinct cells within an individual, derived from a postzygotic mutation. The phenotypes associated with mosaicism depend on the extent of the mosaic cell population. The international BWS consensus group introduced the concept of the Beckwith–Wiedemann spectrum (BWSp) in 2018 (Fig. 4) [12]. Because epigenetic alterations in BWSp are frequently mosaic, variations in the expression of epigenetic alterations in different tissues can lead to the development of clinical features ranging from classic BWS to isolated hemihyperplasia. Similarly, the mosaic tissue distribution of 11p15 epigenetic alterations in SRS can produce various phenotypes, including isolated hemihypoplasia, as a part of Silver–Russell spectrum (SRSp) [8]. Therefore, some of the isolated hemihyperplasia/hypoplasia cases can be caused by epigenetic changes on 11p15 [20]. Considering the association between mosaicism and variations in phenotypes, we hypothesized that the level of alteration in DNA methylation affects the extent of hemihyperplasia /hemihypoplasia, manifesting clinically as the degree of LLD.

In this study, we evaluated the underlying epigenetic alterations and potential epigenotype-phenotype correlations, focusing on LLD, in a group of individuals with isolated hemihyperplasia/hemihypoplasia.

## Results

There were 17 male (57 %) and 13 female (30 %) patients. Twenty-eight patients underwent epiphysiodesis and two patients underwent tibial lengthening at a chronological age of 11.6 ± 1.8 years (range 6.9–14.5 years) and at a bone age of 11.7 ± 1.9 years (range 6.1–15 years). The mean chronological age was not different from the mean bone age (p = 0.876). Methylation-specific multiplex-ligation-dependent probe amplification (MS-MLPA) assay detected epigenetic alterations in 9/30 patients (30 %), and bisulfite pyrosequencing detected in 10/30 patients (33 %; Table 1). All nine patients who had epigenetic alterations identified by MS-MLPA also had consistent results with bisulfite pyrosequencing. In the one patient with SRSp (Hemihypoplasia Patient 2 in Table 1), an epigenetic alteration (maternal UPD pattern) was detected but was not in the result of MS-MLPA (skin sample), but rather the result of bisulfite pyrosequencing (skin sample). The pattern of altered methylation identified by bisulfite pyrosequencing tended to be consistent across the samples tested, but not in all tissue samples from each patient (Additional file 1). No patients showed abnormal single nucleotide polymorphism (SNP) array or *CDKN1C* Sanger sequencing results (n = 20). Of the 10 patients with epigenetic alterations, seven had alterations in both the blood and tissue samples, two had alterations only in the tissue samples, and one had alterations only in the blood sample.

**Table 1:** Clinical features and results of molecular tests of patients (n = 30)

Patient No.	Sex	CA* (*yr*)	BA* (*yr*)	Predicted LLD† (*mm*)	Standardized predicted LLD‡ (%)	Epigenotype	MS-MLPA for 11p15	Bisulfite pyrosequencing		
								DMR1§ (*SD*)	DMR2§ (*SD*)	Methylation difference∥ (*SD*)
*Hemihyperplasia*										
1	F	10.8	11	25	1.8	N	N	− 0.72 (B)	0.74 (B)	0
2	F	11	11	33	2.2	N	N	0.56 (F)	1.82 (F)	2.38
3	M	12.8	12.8	51	3.1	DMR1-GOM	DMR1-GOM	2.63 (F)	− 0.68 (F)	3.31
4	M	14.5	15	26	1.5	N	N	− 1.01 (F)	1.43 (F)	2.44
5	M	13	13.3	20	1.1	N	N	− 0.77 (B)	− 1.67 (B)	0.02
6	M	6.9	6.1	112	8.7	pUPD 11p15	DMR1-GOM & DMR2-LOM	3.55 (F)	− 6.72 (F)	10.27
7	M	14	14	20	1.2	DMR2-LOM	DMR2-LOM	0.39 (B)	− 5.59 (B)	3.1
8	M	8.4	8	43	3.2	N	N	1.53 (S)	− 1.52 (S)	0.71
9	M	14.4	14.2	40	2.5	N	N	0.08 (F)	− 1.51 (F)	1.59
10	M	11.2	12	30	2	N	N	− 0.33 (S)	− 0.78 (S)	0.41
11	M	12.4	11.9	34	2.3	N	N	− 1.39 (S)	− 0.04 (S)	1.33
12	M	10.8	10	40	2.8	N	N	0.94 (S)	− 0.81 (S)	0.86
13	M	11	10.9	22	1.5	N	N	0.31 (M)	− 0.74 (M)	0.36
14	M	14.4	15	24	1.3	DMR1-LOM	DMR1-LOM	− 2.12 (B)	− 0.9 (B)	0.4
15	F	11.1	12	32	2.2	DMR1-LOM	DMR1-LOM	− 2.24 (F)	1.29 (F)	3.53
16	M	12.1	11.8	38	2.5	N	N	− 1.46 (F)	1.69 (F)	3.15
17	M	13.6	13.4	20	1.1	N	N	− 0.72 (B)	1.54 (B)	0.97
18	F	12	12	26	1.6	N	N	− 1.93 (B)	1.91 (B)	2.15
19	F	12.1	12	25	1.6	N	N	− 0.68 (B)	1.58 (B)	1.98
20	F	10.6	11.3	35	2.4	N	N	1.32 (M)	− 1.62 (M)	0.95
21	F	12	11	25	1.6	N	N	− 0.89 (B)	0.89 (B)	0.09
22	F	11.5	11	26	1.7	N	N	−1.12 (S)	1.17 (S)	0.59
23	F	7.9	7.9	61	4.8	DMR2-LOM	DMR2-LOM	− 1.65 (B)	− 14.72 (B)	7.77
*Hemihypoplasia*										
1	M	12	12	43	2.9	N	N	0.97 (M)	− 0.91 (M)	0.99
2	F	10.1	12.1	57	4.1	mUPD 11p15	N	− 2.65 (S)	2.71 (S)	3.27
3	M	11.7	11.8	44	3	DMR2-LOM	DMR2-LOM	− 0.02 (B)	− 3.25 (B)	3.08
4	M	12.2	12	26	1.7	N	N	0.94 (M)	− 1.47 (M)	0.67
5	F	11.4	12	54	3.5	DMR2-LOM	DMR2-LOM	0.69 (F)	− 2.51 (F)	3.2
6	F	11.2	11	20	1.4	DMR1-LOM	DMR1-LOM	− 3.96 (B)	0.18 (B)	2.31
7	M	12.3	12.3	37	2.5	N	N	− 1.65 (S)	1.6 (S)	3.08

*Age at epiphysiodesis or tibial lengthening was presented

†Leg length discrepancy at skeletal maturity was predicted using the multiplier method

‡Predicted leg length discrepancy was divided by the height at the time of epiphysiodesis or tibial lengthening

§Among blood and tissue samples, results of the sample with the maximum methylation difference were described

∥Methylation difference was calculated in fat samples as follows: ∣altered DNA methylation level (SD) at DMR1 − DMR2∣

CA , hronological age; BA, bone age; LLD, leg length discrepancy; MS-MLPA, methylation-specific multiplex-ligation-dependent probe amplification; DMR1, differentially methylated region 1; DMR2, differentially methylated region 2; SD, standard deviation; N, normal; GOM, gain of methylation; LOM, loss of methylation; pUPD, paternal uniparental disomy; mUPD, maternal uniparental disomy; B, blood; F, fat; S, skin; M, muscle

**Fig. 5:**
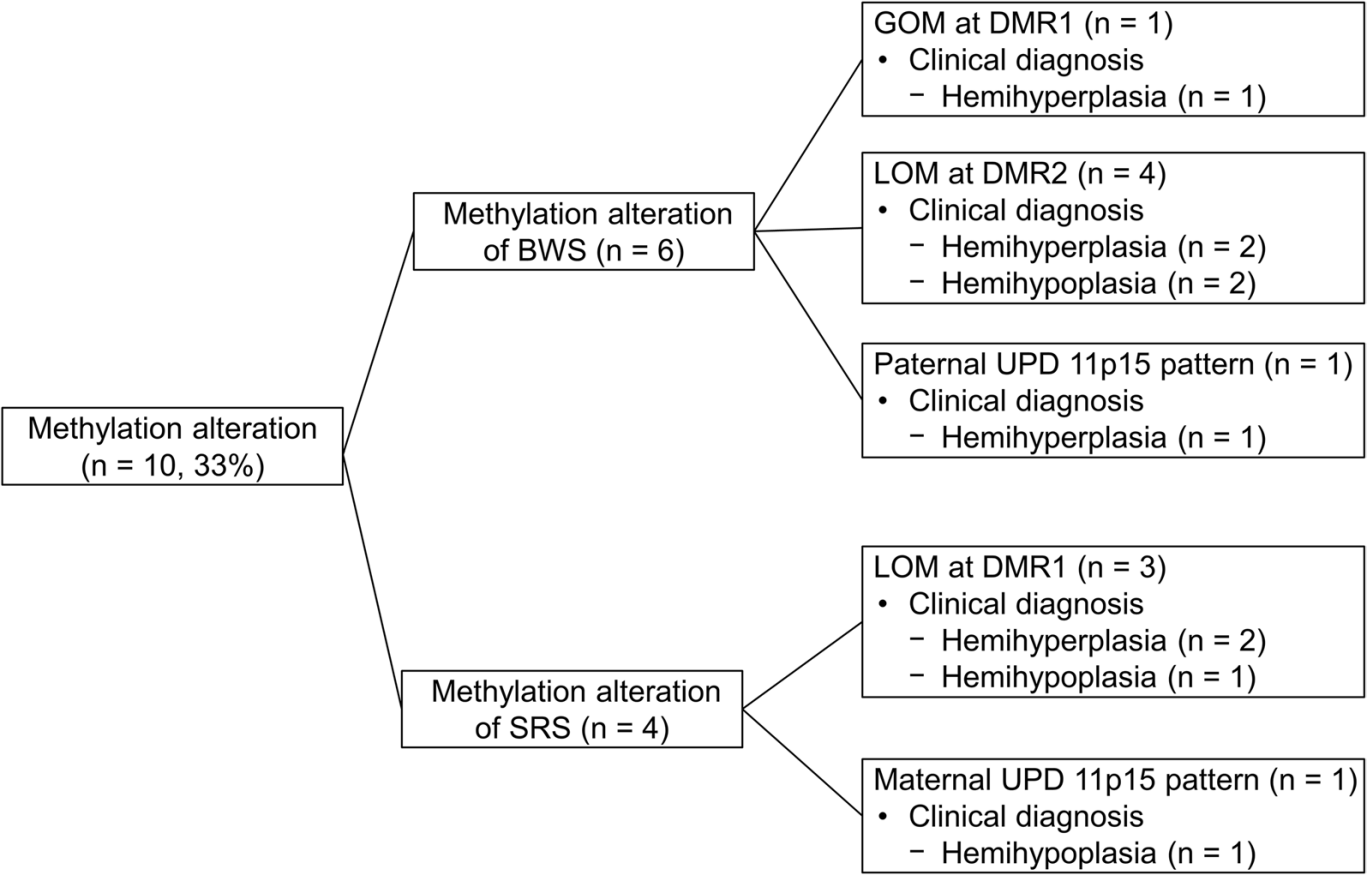
Clinical diagnosis and results of molecular tests of patients

Of the 10 patients with epigenetic alterations, eight had GOM or LOM at DMR1 or DMR2 on 11p15 and two had a UPD 11p15 pattern. No patients had germline mutations in *CDKN1C* or 11p15 copy number variation. Of these 10 patients, six had epigenetic alterations of BWSp and four had alterations of SRSp (Table 1) (Fig. 5). The epigenotypes of six patients with BWSp were LOM at DMR2 in four patients, GOM at DMR1 in one, and paternal UPD 11p15 pattern (both GOM at DMR1 and LOM at DMR2) in one. The epigenotypes of four patients with SRSp were LOM at DMR1 in three patients and maternal UPD 11p15 pattern (both LOM at DMR1 and GOM at DMR2) in one. The clinical diagnosis of six patients with BWSp was hemihyperplasia in four and hemihypoplasia in two. The clinical diagnosis of four patients with SRSp was hemihyperplasia in two and hemihypoplasia in two.

**Fig. 6:**
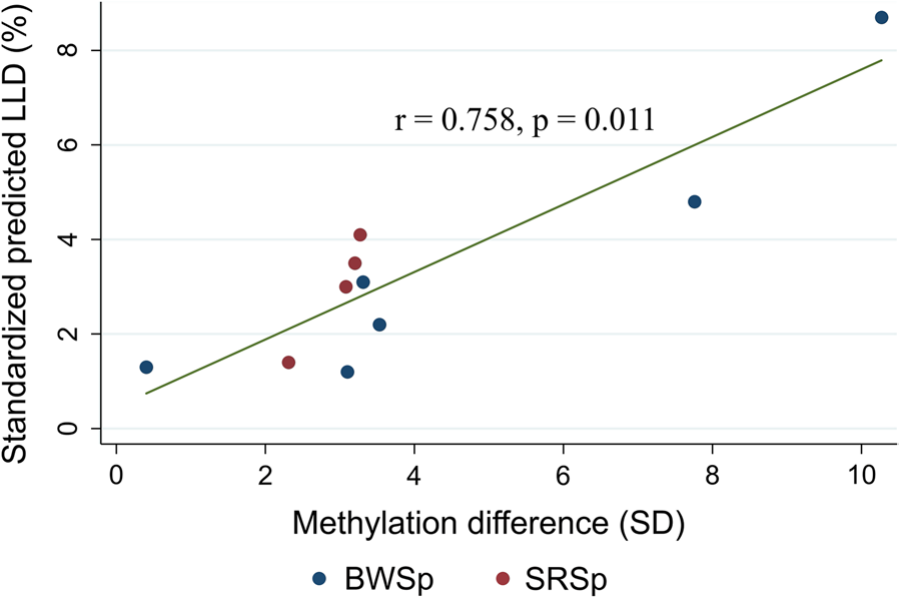
Scatterplot of the methylation difference using fat tissue and predicted leg length discrepancy (LLD) at skeletal maturity in 10 patients with methylation alterations. Blue dots refer to patients with Beckwith–Wiedemann spectrum (BWSp) and red dots refer to patients with Silver–Russell spectrum (SRSp)

Choufani et al. [14] The mean standardized predicted LLD (± standard deviation [SD]) was 2.5 ± 1.5 % (range 1.1–8.7 %). The methylation difference using fat tissue was 2.13 ± 2.23 SD (range 0–10.3 SD) and this showed a moderate correlation with the standardized predicted LLD at skeletal maturity in all patients (r = 0.534; *p* = 0.002) (Table 2) [21]. The methylation difference using fat tissue was strongly correlated with the standardized predicted LLD in 10 patients with methylation alterations (r = 0.758; *p* = 0.011) (Fig. 6). The methylation difference using skin tissue was 2.13 ± 1.98 SD (range 0.12–8.73 SD) and also showed a moderate correlation with the standardized predicted LLD at skeletal maturity in all patients (r = 0.504; *p* = 0.005). However, it showed borderline significance (r = 0.6, *p* = 0.067) in 10 patients with methylation alterations, probably because of a type-II error. The methylation differences using blood and muscle samples were not correlated with the standardized predicted LLD at skeletal maturity.

**Table 2:** Correlations between the methylation difference and predicted LLD

Sample type	Overall patients (n = 30)		Patients with methylation alteration (n = 10)		
	Spearman’s rho	*p* value	Spearman’s rho		*p* value
Blood	0.004	0.985	0.212		0.556
Skin	0.504	0.005	0.600		0.067
Fat	0.534	0.002	0.758		0.011
Muscle	0.057	0.764	0.212		0.556

## Discussion

We demonstrated that isolated hemihyperplasia and hemihypoplasia can occur as BWSp and SRSp, and the methylation difference is related to the predicted LLD at skeletal maturity. This is the first study to identify a relationship between the predicted severity of LLD and epigenetic alterations, providing a basis for understanding the development of idiopathic LLD and value of epigenetic tests for patients with isolated hemihyperplasia/hemihypoplasia.

Several previous studies have shown that patients with isolated hemihyperplasia/hemihypoplasia may have epigenetic alterations that are found in BWSp/SRSp [6, 7, 22–24]. Shuman et al. reported eight patients with paternal UPD 11p15 and three with LOM at DMR2 among 51 patients with isolated hemihyperplasia [7]. Bliek et al. reported a series of eight patients with clinical features ranging from isolated hemihypoplasia to full-spectrum SRS who had LOM at DMR1 [22]. However, all previous studies of “isolated hemihyperplasia/hemihypoplasia” included both patients with isolated hemihyperplasia/hemihypoplasia and patients with skin pigmentation or other BWSp/SRSp clinical features who did not fit the definition of “isolated” [6, 7, 22–24]. Previous studies did not obtain tissue samples from every patient, which may have resulted in false negative results, considering the mosaic distribution of the affected cells. To the best of our knowledge, this is the first study to reveal epigenetic alterations on 11p15 using paired blood-tissue samples in a group of individuals with “pure” isolated hemihyperplasia/hemihypoplasia.

Although *CDKN1C* mutations have been identified in 8 % of patients with BWSp [14], we did not observe these mutations. This may be because the underlying molecular defect of *CDKN1C* mutations is a germline mutation that affects all cells in the body rather than a somatic mosaicism and because our study population comprised isolated hemihyperplasia/hemihypoplasia patients. Previous studies of BWSp/SRSp reported the extreme rarity of hemihyperplasia/hemihypoplasia in patients with *CDKN1C* mutations [16, 20, 25–27]. *CDKN1C* sequencing is therefore thought to be unnecessary in patients with isolated hemihyperplasia/hemihypoplasia.

Because the epigenetic alteration exists in a mosaic form in BWSp/SRSp, it is possible that the proportion of cells with altered methylation is high in hyperplastic/hypoplastic tissues. Therefore, the level of DNA methylation alteration may differ even in patients with the same epigenotype, leading to differences in the phenotype severity. We quantitatively represented this “methylation burden” as the methylation difference between DMR1 and DMR2, which was shown to correlate with the severity of LLD when using skin and fat tissues. Particularly, UPD affects methylation at both DMR1 and DMR2 in opposite directions, resulting in a large methylation difference. In this study, two patients showed a UPD pattern. One patient with a maternal UPD pattern had 57 mm of predicted LLD, and another patient with paternal UPD had 112 mm of predicted LLD. A previous case report described a patient with paternal UPD 11p15 who had hemihyperplasia affecting the right leg and showed 50 mm of LLD at an early age of 8.3 years [28]. Another study also reported that patients with paternal UPD 11p15 had more severe LLD at diagnosis and progression over time than BWSp patients with other molecular defects [29]. Therefore, UPD appears to be associated with severe phenotypes. This is the first study to quantitatively analyze the association between the degree of epigenetic alteration and severity of phenotype in patients with BWSp/SRSp. This strategy may be applicable in patients with BWSp to assess tumor development risk. Correlation analysis using skin tissue might also be better choice than the use of fat tissue for predicting LLD, considering its availability. However, this should be further validated with a larger number of patients with and without methylation alterations.

Whether to perform epigenetic tests using different types of samples on patients with isolated hemihyperplasia/hemihypoplasia could be controversial. If we identify methylation alterations via epigenetic tests, we can reassure patients that the risk of recurrence of hemihyperplasia/hemihypoplasia in their offspring is negligible [19]. If a UPD pattern is observed, we can inform patients of the possibility of lengthening procedures, rather than an operative procedure that suppresses longitudinal bone growth, or epiphysiodesis, for large LLD. In this study, we confirmed epigenetic alterations using peripheral blood samples in 8/10 patients with methylation defect. Therefore, blood sampling only might be adequate as the first step, and multiple tissue sampling could be avoided in most patients with isolated hemihyperplasia and hemihypoplasia for molecular diagnosis of BWSp/SRSp.

Because there are no clinical features other than a size difference between the sides in isolated hemihyperplasia/hemihypoplasia, it is difficult to differentiate these two diseases clinically. Unlike in hemihyperplasia, LLD in hemihypoplasia is rarely more than 25 mm and therefore does not require surgery [3]. However, in this study, the predicted LLDs of two of four patients with the epigenotype of SRSp were 57 and 32 mm. Therefore, LLD due to hemihypoplasia in SRSp can exceed 25 mm, and we should not differentiate these two diseases based on the severity of LLD.

In this study, the clinical diagnosis of hemihyperplasia/hemihypoplasia was not compatible with epigenetic alterations in four of 10 patients. Other studies have also found LOM at DMR1, which should cause hemihypoplasia in patients diagnosed with isolated hemihyperplasia [5, 8]. Because screening for embryonal tumors is recommended only for patients with hemihyperplasia, and not for patients with hemihypoplasia [12, 13], the differentiation of isolated hemihyperplasia from hemihypoplasia has serious prognostic implications. However, without epigenetic tests, this discrimination is nearly impossible, except in a few patients with extreme phenotypes. This problem highlights the need to perform epigenetic tests in patients with isolated hemihyperplasia/hemihypoplasia.

LLD is a common orthopedic condition, which frequently occurs for unknown causes [30]. This study provides a basis for understanding the molecular mechanism underlying idiopathic LLD. When epigenetic alterations of BWSp/SRSp are confined to the thigh and/or leg due to somatic mosaicism, the condition appears as idiopathic LLD. A considerable portion of idiopathic LLD appears to occur as a BWSp/SRSp.

In the present study, tissue samples could be obtained only from the operated legs for ethical reasons. Tibial lengthening was performed only in one of 23 patients with a clinical diagnosis of hemihyperplasia and one of seven patients with clinical diagnosis of hemihypoplasia because the morbidity of tibial lengthening is much greater than that of epiphysiodesis (Additional file 1). Therefore, of 23 patients with a clinical diagnosis of hemihyperplasia, tissue samples were obtained from a longer leg in 22 patients and from a shorter leg in one patient. Of seven patients with a clinical diagnosis of hemihypoplasia, tissue samples were obtained from a shorter leg in one patient and from a longer leg in six patients. Of six patients with BWSp, tissue samples were obtained from an unaffected leg in one patient who underwent tibial lengthening and from an affected leg in the others who underwent epiphysiodesis (Additional file 1). Of four patients with SRSp, tissue samples were obtained from an affected leg in one patient who underwent tibial lengthening and from an unaffected leg in the others who underwent epiphysiodesis. We still do not know whether the tissue samples came from affected or unaffected leg of patients whose epigenetic alterations were not detected. If we could obtain tissue samples from an affected leg in every patient, the detection rate of epigenetic alterations might increase. It is noteworthy that epigenetic alterations were detected in an “unaffected” leg in some patients. Not the presence of epigenetic alteration itself but the difference in the proportion of cells with altered methylation might determine LLD.

This study has several limitations. First, we did not examine other genetic mechanisms affecting growth [31–33]. These mechanisms can be confounding factors that may block the correlation between the methylation difference and predicted LLD. In the first five consecutive patients, we examined genes involved in the PI3K/AKT/mTOR pathway, which is also known to cause syndromic hemihyperplasia [32], using tissue-blood paired high-depth exome sequencing. However, we found no meaningful tissue-specific variant in any sample and thus discontinued the examination. Second, the predicted LLD calculated by the multiplier method may differ from the true LLD [34], although this is one of the most frequently used methods for predicting LLD [35]. Because the development of LLD over time can vary between patients with and without epigenetic alterations [29], patient-specific methods to predict LLD at skeletal maturity based on epigenotypes might be applied. Third, the sample size in this study was small for some statistical analysis, to verify the statistical significance.

## Conclusions

Isolated hemihyperplasia and hemihypoplasia can occur as a spectrum of BWS and SRS. Although the accurate differentiation between isolated hemihyperplasia and isolated hemihypoplasia is important in tumor surveillance planning, it is often difficult to clinically differentiate these two diseases without epigenetic tests. Epigenetic tests may play a role in the prediction of leg length discrepancy, which would aid in treatment planning.

## Methods

### Patients

**Fig. 7:**
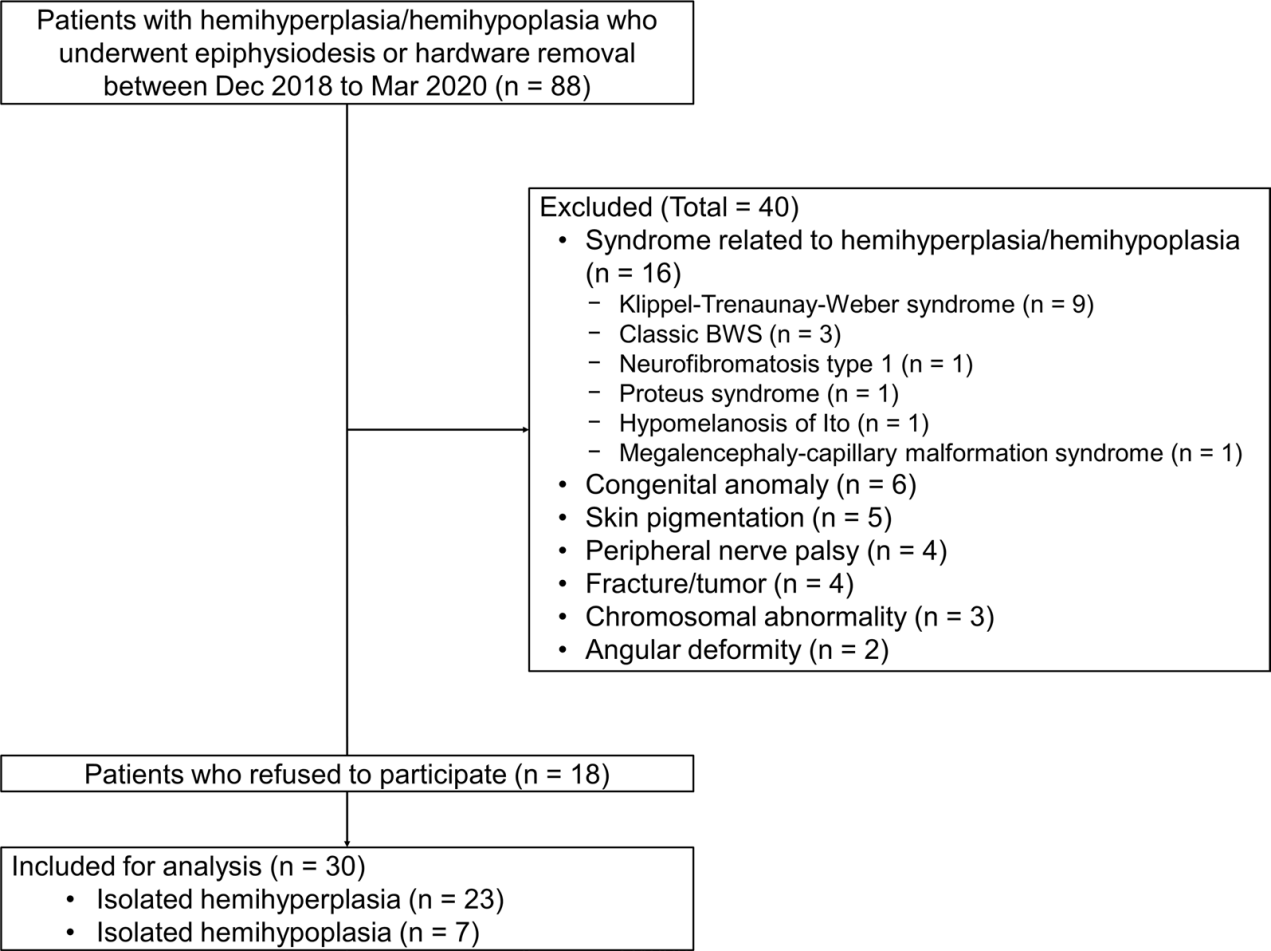
Flowchart of the study population

Eighty-eight patients with hemihyperplasia/hemihypoplasia underwent epiphysiodesis or removal of hardware which had been inserted to correct LLD at a single tertiary-care pediatric center between December 2018 and March 2020 (Fig. 7). Because isolated hemihyperplasia/hemihypoplasia is a diagnosis of exclusion, 40 patients whose hemihyperplasia/hemihypoplasia may have been caused by other medical conditions were excluded following discussions between a pediatric orthopedic surgeon (C.H.S.) and clinical geneticist (J.M.K.). This group included 16 patients with genetic syndromes related to hemihyperplasia/hemihypoplasia, six patients with other congenital anomalies, five patients with skin pigmentation, four patients with peripheral nerve palsy, four patients with a history of fracture or tumor of the lower limbs, three patients with chromosomal abnormalities, and two patients with angular deformity of the lower limbs. We excluded all patients who had one of any phenotypes of BWSp/SRSp other than LLD such as umbilical hernia or oligohydramnios to confine the study population to patients with isolated hemihyperplasia/hemihypoplasia. Of the remaining 48 patients, 18 patients (38%) refused to participate. Twenty-three patients with hemihyperplasia and seven patients with hemihypoplasia constituted the study group. Hemihyperplasia and hemihypoplasia were classified by each surgeon preoperatively based on normative height, sitting height, and subischial leg length data [36, 37].

**Fig. 8:**
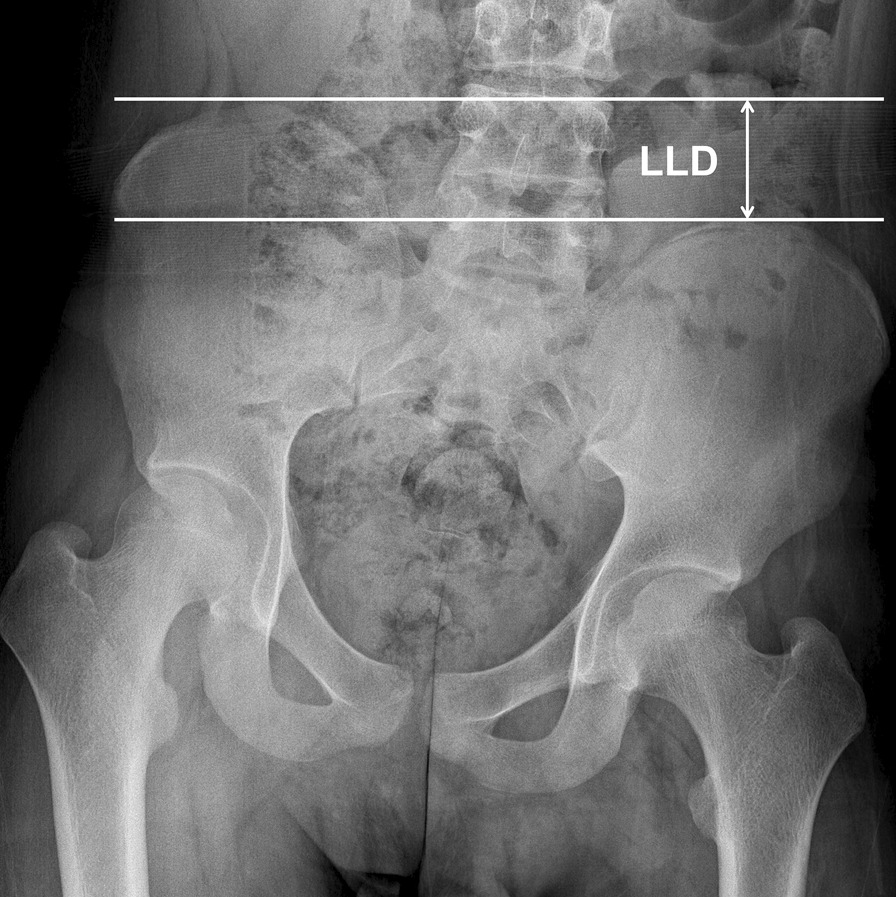
Measurement of leg length discrepancy (LLD) on a standing pelvic anteroposterior radiograph

Bone age was estimated using the Greulich-Pyle atlas method [38]. LLD was measured as the iliac crest height difference on a standing pelvic anteroposterior radiograph (Fig. 8). LLD at skeletal maturity was predicted using a multiplier method at the time of epiphysiodesis or tibial lengthening [39]. Chronological age was used for a multiplier method according to its original description [39]. To standardize the predicted LLD for stature, the predicted LLD was divided by the height at the time of epiphysiodesis or tibial lengthening.

We introduced a metric named the methylation difference, defined as the difference in DNA methylation levels between DMR1 and DMR2 in the same tissue using bisulfite pyrosequencing. Because GOM at DMR1 promotes growth via transcription of *IGF2* and GOM at DMR2 restricts growth via transcription of *CDKN1C* [14], the methylation difference would determine the direction of growth. We assumed that the methylation difference is correlated with the severity of predicted LLD at skeletal maturity.

### Sample collection

Because of somatic mosaicism, 11p15 epigenetic alterations in individual patients may differ between cells from different tissues, and acquisition of tissue samples from overgrown/undergrown regions increases the likelihood of finding epigenetic alterations [7]. We usually perform a molecular test using blood samples as a first line test and consider an additional test using skin or other tissue samples when the blood analysis is negative. Because patients in the present study were supposed to undergo surgery for LLD, we decided to obtain blood and tissue samples at the same time under general anesthesia without causing additional pain from blood sampling. Skin biopsy samples have been preferred because of their high accessibility, relative to other organs, in previous studies [40]. Because we hypothesized that local tissue that had developed from the mesoderm would contribute more to leg length than peripheral blood or local tissue from the ectoderm, we obtained fat and muscle tissues, as well as skin tissue, to increase the rate of molecular detection. Therefore, at the time of operation, we collected paired blood-tissue samples composed of 5 mL of peripheral blood and a small amount of dermis, fat, and muscle. Tissue samples were obtained through the incision made for epiphysiodesis or hardware removal. Muscles were obtained from the vastus lateralis using a 3-mm punch in 17 patients undergoing operations at the distal femur and proximal tibia and in 11 patients at the distal femur, and from the tibialis anterior in two patients undergoing operations at the tibia. The mean age of the patients was 12.9 ± 1.8 years (range 7.9–15.7 years) at the time of sample acquisition.

### Molecular testing

**Fig. 9:**
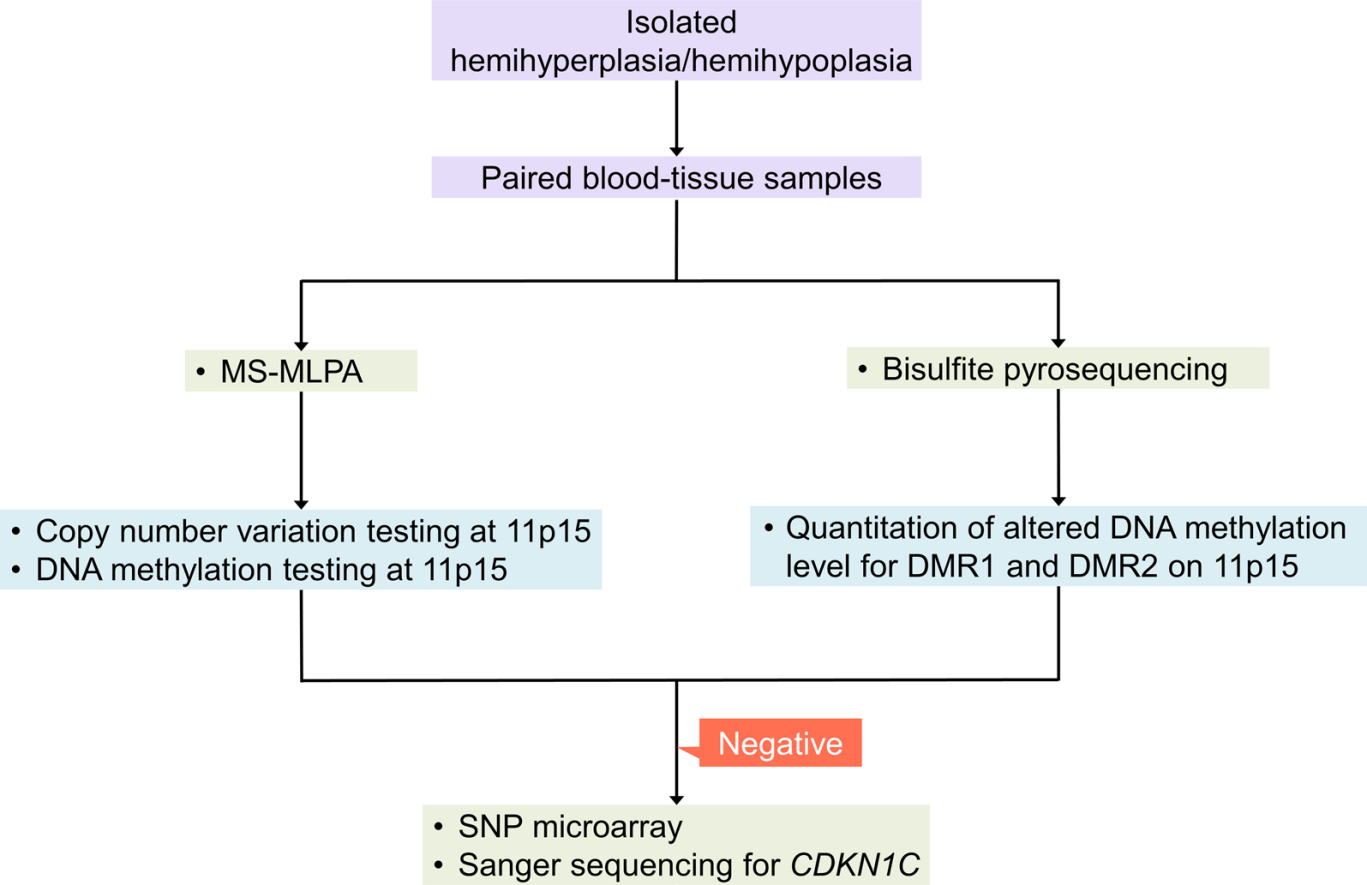
Flowchart of the molecular test

Molecular testing to identify the genetic and epigenetic alterations responsible for BWSp/SRSp, (1) GOM or LOM at DMR1 or DMR2 on 11p15, (2) UPD 11p15, (3) germline mutations in *CDKN1C*, or (4) 11p15 copy number variation [14–19], was performed. MS-MLPA assay and bisulfite pyrosequencing for DMR1 and DMR2 on 11p15 were performed for all patients (Fig. 9). MS-MLPA can detect methylation alteration, UPD, and copy number variation, and bisulfite pyrosequencing can detect methylation alteration and UPD. Samples from patients who did not show abnormalities of any tissue in MS-MLPA and bisulfite pyrosequencing (n = 20) were further analyzed by SNP microarray which can detect UPD and copy number variation and *CDKN1C* Sanger sequencing. We did not perform SNP array for the patients who showed UPD 11p15 pattern in methylation studies. Low-grade tissue mosaicism of UPD or short range (< 10 MB) loss of heterozygosity cannot be detected and reported based on the SNP microarray platform used in the present study (Affymetrix CytoScan 750 K Array [Thermo Fisher Scientific, Walsham, United States]). The details of molecular testing are shown in Additional file 2.

### Statistical analysis

The mean chronological age was compared to the mean bone age using the Wilcoxon signed-rank test after the Shapiro-Wilks test for normality. The methylation difference was calculated as ∣altered DNA methylation level (SD) at DMR1 − altered DNA methylation level (SD) at DMR2∣ in the same tissue using bisulfite pyrosequencing. Methylation differences were calculated in the same way regardless of patients’ epigenotypes. The correlation between the methylation difference and standardized predicted LLD was evaluated using Spearman correlation test. Significance was set at *p* < 0.05. Statistical analysis was performed using STATA 15.1 (Stata Corp, College Station, TX, USA).

## Supplementary Information

**Additional file 1: Supplementary Table 1**. Results of bisulfite pyrosequencing.

**Additional file 2**. Additional details of molecular testing.

## Data Availability

The datasets used and analyzed during the current study are available from the corresponding author on reasonable request.

## Abbreviations

BWS: Beckwith–Wiedemann syndrome.
BWSp: Beckwith–Wiedemann spectrum.
DMR1: Differentially methylated region 1.
DMR2: Differentially methylated region 2.
GOM: Gain of methylation.
LLD: Leg length discrepancy.
LOM: Loss of methylation.
MS-MLPA: Methylation-specific multiplex-ligation-dependent probe amplification assay.
SD: standard deviation.
SNP: Single nucleotide polymorphism.
SRS: Silver–Russell syndrome.
SRSp: Silver–Russell spectrum.
UPD: Uniparental disomy
