# Supplementary material for: Prospective study of epigenetic alterations responsible for isolated hemihyperplasia/hemihypoplasia and their association with leg length discrepancy
Source: Orphanet J Rare Dis. 2021 Oct 9;16:418. doi: 10.1186/s13023-021-02042-6 (PMC8501601; doi:10.1186/s13023-021-02042-6)
Supplement: Supplementary file 1 — Additional file 1: Supplementary Table 1. Results of bisulfite pyrosequencing. [file 13023_2021_2042_MOESM1_ESM.docx]

**Additional File 1.**

| Supplementary Table 1. Results of bisulfite pyrosequencing | | | | | | | | | | | | | | |  |
| --- | --- | --- | --- | --- | --- | --- | --- | --- | --- | --- | --- | --- | --- | --- | --- |
| Patient No. | Blood | |  | Skin | |  | Fat | | |  | Muscle | |  | Operations performed | |
|  | DMR1 (SD) | DMR2 (*SD*) |  | DMR1 (*SD*) | DMR2 (*SD*) |  | | DMR1 (*SD*) | DMR2 (*SD*) |  | DMR1 (*SD*) | DMR2 (*SD*) |  |  |  |
| Hemihyperplasia | | | | | | | | | | | | | | |  |
| 1^*^ | −0.72 | 0.74 |  | −0.53 | −0.89 |  | | −0.86 | −0.84 |  | −0.73 | −1.55 |  | Epiphysiodesis | |
| 2^*^ | −0.89 | −0.91 |  | −0.43 | 1.04 |  | | 0.56 | 1.82 |  | −0.42 | 0.38 |  | Epiphysiodesis | |
| 3^†^ | 2.09 | −0.1 |  | 1.92 | 0 |  | | 2.63 | −0.68 |  | 2.2 | 1.36 |  | Epiphysiodesis | |
| 4^*^ | −0.84 | 1.43 |  | −0.71 | −0.83 |  | | −1.01 | 1.43 |  | −0.68 | −0.26 |  | Epiphysiodesis | |
| 5^*^ | −0.77 | −1.67 |  | −0.4 | −0.22 |  | | −0.92 | −0.94 |  | −0.71 | 0.12 |  | Epiphysiodesis | |
| 6^‡^ | 3.07 | −3.21 |  | 2.06 | −4.59 |  | | 3.55 | −6.72 |  | 2.5 | −4.29 |  | Tibial lengthening | |
| 7^†^ | 0.39 | −5.59 |  | 0.38 | −2.09 |  | | 0.67 | −2.43 |  | 0.33 | −2.36 |  | Epiphysiodesis | |
| 8^*^ | 0.56 | 1.09 |  | 1.53 | −1.52 |  | | 1.71 | 1 |  | 1.31 | 0.79 |  | Epiphysiodesis | |
| 9^*^ | −0.94 | −0.58 |  | −0.14 | 0.82 |  | | 0.08 | −1.51 |  | −0.55 | −0.63 |  | Epiphysiodesis | |
| 10^*^ | −0.32 | −0.54 |  | −0.33 | −0.78 |  | | −0.35 | −0.76 |  | −0.41 | −0.84 |  | Epiphysiodesis | |
| 11^*^ | −0.91 | −1.42 |  | −1.39 | −0.04 |  | | −1.27 | 0.06 |  | −1.22 | 0.01 |  | Epiphysiodesis | |
| 12^*^ | −0.95 | −0.34 |  | 0.94 | −0.81 |  | | −0.11 | −0.97 |  | −0.7 | −0.8 |  | Epiphysiodesis | |
| 13^*^ | −0.9 | 0.1 |  | −0.9 | −0.19 |  | | 0.29 | −0.07 |  | 0.31 | −0.74 |  | Epiphysiodesis | |
| 14^‡^ | −2.12 | −0.9 |  | −1.71 | −0.93 |  | | −1.43 | −1.03 |  | −1.09 | 0.01 |  | Epiphysiodesis | |
| 15^‡^ | −3.25 | −1.19 |  | −2.91 | 0.1 |  | | −2.24 | 1.29 |  | −1.43 | 0.9 |  | Epiphysiodesis | |
| 16^*^ | −1.12 | 1.16 |  | −0.88 | 1.83 |  | | −1.46 | 1.69 |  | −0.21 | 0.63 |  | Epiphysiodesis | |
| 17^*^ | −0.72 | 1.54 |  | −0.43 | 1.02 |  | | 1.05 | 2.02 |  | −0.11 | 1.77 |  | Epiphysiodesis | |
| 18^*^ | −1.93 | 1.91 |  | −1.77 | 1.24 |  | | −1.15 | 1 |  | −0.45 | 0.88 |  | Epiphysiodesis | |
| 19^*^ | −0.68 | 1.58 |  | −1.01 | 0.01 |  | | −0.99 | 0.98 |  | 1.23 | 1.6 |  | Epiphysiodesis | |
| 20^*^ | 1.03 | −0.31 |  | 0.87 | 0.52 |  | | 1.91 | 0.96 |  | 1.32 | −1.62 |  | Epiphysiodesis | |
| 21^*^ | −0.89 | 0.89 |  | 0.73 | −0.1 |  | | 1.51 | 1.42 |  | 0 | −1.6 |  | Epiphysiodesis | |
| 22^*^ | −0.78 | −0.46 |  | −1.12 | 1.17 |  | | −0.56 | 0.03 |  | −1.34 | −0.15 |  | Epiphysiodesis | |
| 23^†^ | −1.65 | −14.72 |  | −1.24 | −9.97 |  | | −1.44 | −9.2 |  | −1.19 | −7.91 |  | Epiphysiodesis | |
| Hemihypoplasia | | | | | | | | | | | | | | |  |
| 1^*^ | −1.95 | −0.59 |  | 0.01 | 1.66 |  | | −1.1 | −0.11 |  | 0.97 | −0.91 |  | Epiphysiodesis | |
| 2^†^ | −1.07 | 0.43 |  | −2.65 | 2.71 |  | | −1.9 | 1.37 |  | 0.5 | −0.26 |  | Tibial lengthening | |
| 3^†^ | −0.02 | −3.25 |  | 0.33 | −2.43 |  | | −0.07 | −3.15 |  | 0.19 | −0.79 |  | Epiphysiodesis | |
| 4^*^ | 0.1 | 0.46 |  | 0.44 | 0.59 |  | | 0.58 | −0.09 |  | 0.94 | −1.47 |  | Epiphysiodesis | |
| 5^†^ | −0.33 | −1.12 |  | −0.52 | −1.76 |  | | 0.69 | −2.51 |  | 0.05 | −2.36 |  | Epiphysiodesis | |
| 6^‡^ | −3.96 | 0.18 |  | −3.25 | 0.54 |  | | −2.94 | −0.63 |  | −1.86 | −0.49 |  | Epiphysiodesis | |
| 7^*^ | −0.88 | 0.99 |  | −1.65 | 1.6 |  | | −1.54 | 1.54 |  | −1.98 | −0.05 |  | Epiphysiodesis | |
| *It was unclear whether tissue samples were obtained from the affected or unaffected leg. †Tissue samples were obtained from the affected leg. ‡Tissue samples were obtained from the unaffected leg. DMR1 = differentially methylated region 1, DMR2 = differentially methylated region 2, and SD = standard deviation | | | | | | | | | | | | | | |  |
|  | | | | | | | | | | | | | | |  |
