# Supplementary material for: Prospective study of epigenetic alterations responsible for isolated hemihyperplasia/hemihypoplasia and their association with leg length discrepancy
Source: Orphanet J Rare Dis. 2021 Oct 9;16:418. doi: 10.1186/s13023-021-02042-6 (PMC8501601; doi:10.1186/s13023-021-02042-6)
Supplement: Supplementary file 2 — Additional file 2. Additional details of molecular testing. [file 13023_2021_2042_MOESM2_ESM.docx]

**Additional File 2.**

*Additional details of molecular testing*

Additional details on the methods used in this study are described below.

**Methylation-specific multiplex-ligation-dependent probe amplification (MS-MLPA)**

MS-MLPA assay can simultaneously detect copy number variations in the target region and DNA methylation alterations [1]. The DNA copy number changes and methylation pattern on chromosome 11p15 were first investigated via MS-MLPA using the SALSA MLPA kit, ME030 BWS/RSS (MRC Holland, Amsterdam, Netherlands), according to the manufacturer’s instructions. Probes were hybridized to denatured genomic DNA, and hybridized samples were divided into two separate tests: one ligated directly and the other digested with HhaI, an MS restriction enzyme, before ligation. After ligation, PCR was carried out with fluorescence-labeled unique primers for the probe sets, including differentially methylated regions 1 and 2 (DMR1 and DMR2). The former identifies copy number changes of CDKN1C, KCNQ1, KCNQ1OT1, IGF2, and H19, whereas the latter determines the methylation status of DMR1 and DMR2 on 11p15. Amplified products were separated on an ABI3130xl Genetic Analyzer (Applied Biosystems, Foster City, CA, USA) and analyzed using the GeneMarker v.1.9 (SoftGenetics, Pittsburgh, PA, USA), with at least three control samples for each test.

**Bisulfite pyrosequencing**

Bisulfite pyrosequencing provides quantitative values for methylation alterations and increases the diagnostic yield, particularly in patients with mild and marginal methylation alterations [2]. Bisulfite pyrosequencing was performed for DMR1 and DMR2 using targeted assays covering seven consecutive CpG sites for DMR2 and five CpG sites for DMR1. Sodium bisulfite modified genomic DNA was amplified using Hot-Start Taq master mix (Qiagen, Hilden, Germany). Regions of interest were amplified by PCR, and pyrosequencing was carried out using the PyroMark Q24 pyrosequencer (Qiagen) according to the manufacturer's protocol. Output data were analyzed using PyroMark Q24 1.0.10 Software (Qiagen), which calculates the methylation percentage (mC/(mC + C)) for each CpG site, allowing quantitative comparisons. Altered DNA methylation levels (%mC) at DMR1/DMR2 were calculated using the average DNA methylation detected in 20 samples from Korean children as controls. The gain of methylation and loss of methylation were defined based on the DNA methylation level detected in the patient sample ± 2 SD from the control mean [3]. Reference values (% mC, ± 2 SD) were as follows: DMR1 (49.1–59.7) and DMR2 (56.4–69.9). Primers used for pyrosequencing analyses are listed in Supplementary Table 2.

**Single nucleotide polymorphism** (**SNP) microarray**

SNP microarray is a sensitive method for detecting uniparental disomy, larger copy number variations, and low-level mosaicism of approximately 15–20% throughout the whole genome [4]. Therefore, SNP microarray can help rule out chromosomal aberrations comprising 11p15 producing body asymmetry due to somatic mosaicism. The Affymetrix CytoScan 750K Array (Affymetrix, Santa Clara, CA, USA) was used. Following the manufacturer’s protocol, 50 ng/μL of genomic DNA from each patient was digested with the restriction enzyme Nsp I. Next, the DNA was ligated to a common adaptor using T4 DNA ligase. Following ligation, the template was subjected to PCR amplification using Titanium Taq DNA polymerase. The amplified PCR product was pooled and purified using bead-based purification methods. The purified product was quantified and fragmented with Fragmentation Reagent (DNase I) and end-labeled using terminal deoxynucleotidyl transferase. The labeled samples were hybridized by loading on arrays. After hybridization, the arrays were washed, stained, and scanned. The data were analyzed using the software Chromosome Analysis Suite (Affymetrix). The Database of Genomic Variants (GRCh37/ hg19) and OMIM, DECIPHER were used to evaluate the array data and analyze genotype-phenotype correlations.

**Sanger sequencing for *CDKN1C***

Because BWS and SRS can occur via *CDKN1C* loss-of-function mutations and gain-of-function mutations on the maternal allele [5, 6], *CDKN1C* Sanger sequencing was performed. Sanger sequencing of *CDKN1C* (NM_000076) was performed to rule out the presence of point mutations in *CDKN1C*. Two coding *CDKN1C* exons and exon–intron boundaries were amplified by PCR and directly sequenced using an ABI3130x1 Genetic Analyzer (Applied Biosystems). Primers used for *CDKN1C* sequencing analyses are listed in Supplementary Table 3.

| **Supplementary** **Table 2.** Primers used for bisulfite pyrosequencing | | | |
| --- | --- | --- | --- |
| Gene | Forward Primer (5' to 3') | Reverse Primer (5' to 3') | Sequencing Primer (5' to 3') |
| DMR1 (*H19/IGF2*) | TGAGTGTTTTATTTTTAGATGATTTT | GTTGTGATGTGTGAGTTTGTATTGT | GTGGTTTGGGTGATT |
| DMR2 (*KCNQ10T1*) | GTGATGTGTTTATTATT | TGGAGGTTTGTGGGYGTTTAG | GTGATGTGTTTATTATT |
| DMR1 = differentially methylated region 1 and DMR2 = differentially methylated region 2 | | | |

**Supplementary** **Table 3.** Primers used for *CDKN1C* Sanger sequencing

| *CDKN1C* | Forward Primer (5' to 3') | Reverse Primer (5' to 3') | Size (bp) |
| --- | --- | --- | --- |
| Exon1_1 | CGTTCCACAGGCCAAGTGCG | GCTGGTGCGCACTAGTACTG | 374 |
| Exon1_2 | CGTCCCTCCGCAGCACATCC | CCTGCACCGTCTCGCGGTAG | 279 |
| Exon1_3 | TGGACCGAAGTGGACAGCGA | AGTGCAGCTGGTCAGCGAGA | 496 |
| Exon1_4 | CCGGAGCAGCTGCCTAGTGTC | CTTTAATGCCACGGGAGGAGG | 539 |
| Exon2 | CGGCGACGTAAACAAAGCTG | GGTTGCTGCTACATGAACGG | 469 |

**References**

1. Priolo M, Sparago A, Mammi C, Cerrato F, Lagana C, Riccio A. MS-MLPA is a specific and sensitive technique for detecting all chromosome 11p15.5 imprinting defects of BWS and SRS in a single-tube experiment. Eur J Hum Genet. 2008;16(5):565-71.

2. Lee BH, Kim GH, Oh TJ, Kim JH, Lee JJ, Choi SH, et al. Quantitative analysis of methylation status at 11p15 and 7q21 for the genetic diagnosis of Beckwith-Wiedemann syndrome and Silver-Russell syndrome. J Hum Genet. 2013;58(9):604-10.

3. Cytrynbaum C, Chong K, Hannig V, Choufani S, Shuman C, Steele L, et al. Genomic imbalance in the centromeric 11p15 imprinting center in three families: Further evidence of a role for IC2 as a cause of Russell-Silver syndrome. Am J Med Genet A. 2016;170(10):2731-9.

4. Russo S, Calzari L, Mussa A, Mainini E, Cassina M, Di Candia S, et al. A multi-method approach to the molecular diagnosis of overt and borderline 11p15.5 defects underlying Silver-Russell and Beckwith-Wiedemann syndromes. Clin Epigenetics. 2016;8:23.

5. Brioude F, Netchine I, Praz F, Le Jule M, Calmel C, Lacombe D, et al. Mutations of the imprinted CDKN1C gene as a cause of the overgrowth Beckwith-Wiedemann syndrome: clinical spectrum and functional characterization. Hum Mutat. 2015;36(9):894-902.

6. Brioude F, Oliver-Petit I, Blaise A, Praz F, Rossignol S, Le Jule M, et al. CDKN1C mutation affecting the PCNA-binding domain as a cause of familial Russell Silver syndrome. J Med Genet. 2013;50(12):823-30.
